# Fatty Acid Based Microemulsions to Combat Ophthalmia Neonatorum Caused by *Neisseria gonorrhoeae* and *Staphylococcus aureus*

**DOI:** 10.3390/nano8010051

**Published:** 2018-01-19

**Authors:** Ummara Butt, Amr ElShaer, Lori A. S. Snyder, Ali A. Al-Kinani, Adam Le Gresley, Raid G. Alany

**Affiliations:** 1 Drug Discovery, Delivery and Patient Care (DDDPC), School of Life Sciences, Pharmacy and Chemistry, Kingston University, Penrhyn Road, Kingston upon Thames, Surrey KT1 2EE, UK; k1266883@kingston.ac.uk (U.B.); A.Alkinani@kingston.ac.uk (A.A.A.-K.); A.Legresley@kingston.ac.uk (A.L.G.); R.Alany@kingston.ac.uk (R.G.A.); 2 School of Life Sciences, Pharmacy and Chemistry, Kingston University, Kingston upon Thames KT1 2EE, UK; L.Snyder@kingston.ac.uk; 3 School of Pharmacy, University of Auckland, Auckland 1142, New Zealand

**Keywords:** fatty acid, ophthalmia neonatorum, microemulsion, pseudo-ternary phase diagram, ocular toxicity/irritation potential

## Abstract

The bacterial species *Neisseria gonorrhoeae* (*N. gonorrhoeae*) and *Staphylococcus aureus* (*S. aureus*) are amongst the main microorganisms that cause ophthalmia neonatorum. The current treatment involves the use of various antibiotics such as ciprofloxacin, cephalosporin, ceftriaxone and cefotaxime. However, this treatment strategy is becoming more ineffective due to the antibiotic resistance in *N. gonorrhoeae*. The current study explores the potential use of fatty acid based microemulsions (ME) to prevent *N. gonorrhoeae* and *S. aureus* infections in new-borns’ eyes without harmful side effects such as corneal or conjunctiva irritation. Pseudo-ternary phase diagrams were constructed to evaluate microemulsion regions and six different α-linolenic acid based microemulsions were prepared. The prepared formulations were characterized for α-linolenic acid content, size, transparency, zeta potential, Polarized light Microscopy, antimicrobial activity and ex vivo ocular toxicity. The mean droplet size of the ME formulations was in the range of 190.4 to 350.5 nm and polydispersity index (PDI) values were in the range of 0.102 to 0.561. All formulations were found stable upon storage for at least 8 weeks. In addition, self-diffusion coefficients determined by nuclear magnetic resonance (NMR) reflected that the diffusability of water increased at higher than 30% *w*/*w* water, while that of fatty acids and surfactants was in reverse. The antimicrobial efficacy of microemulsions was determined against *N. gonorrhoeae* and *S. aureus*. It was concluded that all microemulsions have strong antimicrobial effects against *N. gonorrhoeae* and *S. aureus*. Finally, bovine corneal opacity permeability (BCOP) and hen’s egg chorioallantoic (HET-CAM) tests results showed that all microemulsion formulations were not strong ocular irritants.

## 1. Introduction

Ophthalmia neonatorum is a form of conjunctivitis in new-borns, usually contracted during birth from passage through the infected birth canal of the mother [1]. The bacterial species *Neisseria gonorrhoeae* is the cause of the sexually transmitted disease gonorrhoea [2] and the cause of ophthalmia neonatorum. *Neisseria gonorrhoeae* (*N. gonorrhoeae*) accounts for 30% to 45% of ophthalmia cases [3,4]. Nonsexually transmitted bacteria, such as *Staphylococcus aureus* (*S. aureus*), can also cause neonatal conjunctivitis. *S. aureus* accounts for 30% to 50% cases of ophthalmia neonatorum [3,4]. *N. gonorrhoeae* infection is of particular concern as it cannot be successfully treated, it can cause corneal ulceration and perforation of the globe of the eye, which can rapidly lead to blindness. If left untreated, the neonatal bacterial eye infections can potentially spread, causing life threatening conditions such as septicaemia and meningitis. Opthalmia neonatorum occurs in 1% to 12% of new-born infants and leads to blindness in approximately 10,000 babies annually worldwide [5].

There are a variety of treatments that can be used against gonococcal ophthalmia neonatorum (GCON). Control strategies include the treatment of gonococcal infection in pregnant women, diagnosis and treatment of GCON and eye prophylaxis in the new born at birth. The treatment of gonococcal infections in pregnant women requires screening and is expensive [5]. Eye prophylaxis by the instillation of 1% silver nitrate in the first h of life is not always possible and no longer in use as silver nitrate is toxic and causes chemical conjunctivitis [6,7]. Recently, various antibiotics such as ceftriaxone IV or IM and cefotaxime IM are recommended as first line therapy for gonococcal ophthalmia neonatorum and other gonococcal infections in the new born [8,9]. However, in the last few years, mutants of gonococci expressing significant resistance to these antibiotics have been identified worldwide [10,11].

It has been proposed that a fatty acid based eye formulation can be used as an alternative control strategy [8]. Several fatty acids have a broad spectrum of microbicidal activity against enveloped viruses and various Gram positive and Gram negative bacteria, including *N. gonorrhoeae* and *S. aureus* [8,12]. In recent years, microbicidal effects of medium- and long-chain fatty acids and their corresponding 1-monoglycerides have been studied. They have been found to have a broad spectrum of microbicidal activity against enveloped viruses and various bacteria in vitro, including pathogens such as herpes simplex virus (HSV), *Neisseria gonorrhoea* and *Chlamydia trachomati* [8,12].

Nearly 10 years ago, the bactericidal potencies of saturated and unsaturated fatty acids and monoglycerides (MGs) against *Helicobacter pylori* were determined following short incubations with freshly harvested cells over a range of pHs. Lauric acid was found to kill the bacterial species at a minimum bactericidal concentration (MBC) of 1 mM at pH 7.4, myristoleic and linolenic acid were found to be potent at MBC of 0.5 mM at pH 7.4 and monolaurin was found to be the most potent monoglyceride (MBC 0.5 mM). The bactericidal potencies of unsaturated fatty acids were found to increase with degree of unsaturation [13]. It has been found that *N. gonorrhoeae* is highly susceptible to lauric acid (C12:0), myristic acid (C14:0), oleic acid (C18:1), linoleic acid (C18:2), linolenic acid (C18:3) and arachidonic acid (C20:4) but is resistant to straight-chain, saturated fatty acids with 18 or more carbons [14]. Various long-chain polyunsaturated fatty acids (LC-PUFAs)—including eicosapentaenoic acid (EPA; C20:5n-3), docosahexaenoic acid (DHA; C22:6n-3), γ-linolenic acid (GLA; C18:3n-6) and dihomo-γ-linolenic acid (DGLA; C20:3n-6)—have shown to exert highly potent activity against *S. aureus* [15]. In 1990, Bergsson et al. tested several fatty acids and their 1-monoglycerides for their microbicidal activities against *N. gonorrhoea* at a short inactivation time of 1 min. It was shown that 1 min exposure to 2.5 mM lauric acid and monocaprin—a monoglyceride of capric acid—causes the fastest and most effective killing of all strains of *N. gonorrhoea* tested [8]. Over ten years ago, a hydrogel containing 10 mM monocaprin was found to kill high titres of *N. gonorrhoea* within 1 minute [16]. Although many studies looked at the microbial effects of fatty acids on various microbes, no studies evaluated the effect of fatty acid formulations on bacteria, fungi or viruses.

In the present study, a fatty acid based microemulsions were prepared. α-linolenic acid was selected because of its significant antimicrobial activity against *N. gonorrhoeae* and *S. aureus* as per our previous studies [17,18]. The prepared microemulsions (ME) formulations were characterized by polarized light microscopy and self-diffusion NMR (Nuclear magnetic resonance). Different pharmaceutical formulation properties such as clarity, pH, particle size, viscosity, physical and chemical stability and sterility of the formulations were investigated as part of the development phase of an eye formulation. In addition, antibacterial activity of the α-linolenic acid-based ME was tested against *N. gonorrhoeae* and *S. aureus*. Finally, the eye formulations were tested for ocular toxicity/irritation potential using the bovine corneal opacity and permeability (BCOP) test and the hens egg chorioallantoic membrane test (HET-CAM). The estimated costs for routine neonatal ocular prophylaxis are $7.7 per child and $1.94 for treatment using erythromycin. Nonetheless the costs will increase with the development of resistance to the current antibiotics used for treatment of ophthalmia neonatorum conditions. The use of fatty acids can minimize the costs associated with the antimicrobial resistance [19].

## 2. Results and Discussion

### 2.1. Saturation Solubility

Absorbance was measured by UV spectrophotometer at 350 nm to determine the solubility of α-linolenic acid in various surfactants. The correlation between concentration (mg/mL) and absorbance (average of 3 determinations) was plotted to find out saturation point of a compound. It was found that all excipients were able to dissolve α-linolenic acid, although not to the same extent.

**Figure 1:**
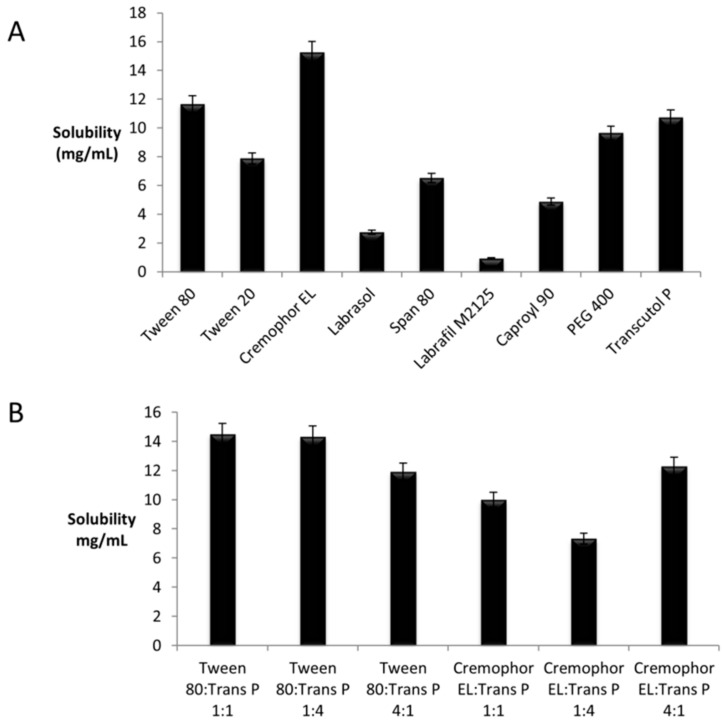
Saturation solubility of α-linolenic acid in different surfactant (**A**) and different ratios of surfactants blends (**B**). Mean ± SD, *n* = 3.

Solubility of α-linolenic acid in different excipients is summarized in Figure 1A. The results of saturation solubility studies revealed that α-linolenic acid has high solubility in Cremophor EL and Tween 80 as compared to Tween 20, Labrasol, Labrafil M2125 and Caproyl 90. Amongst the tested co-surfactants, Transcutol P and PEG 400 solubilized α-linolenic acid the most. Therefore, Tween 80 and Cremophor EL were selected as surfactants and Transcutol P and PEG 400 were selected as cosurfactants, respectively, for the phase behaviour study.

The solubility profile of α-linolenic acid in the selected surfactant/co-surfactant blends as shown in Figure 1B reveals that α-linolenic acid had the highest solubility in Tween 80/Transcutol P mixture compared with Cremophor EL/Transcutol P.

The high solubility of α-linolenic acid in Tween 80 and Cremophor EL could be due to the structural similarities between α-linolenic acid, Tween 80 and Cremophor EL. α-linolenic acid is a long chain (C18) of unsaturated fatty acid. Tween 80 is structurally composed of polyoxyethylene sorbitan (head group) and unsaturated oleic acid (tail group). Tween 80 shares a carbon chain tail of similar length (C18) to α-linolenic acid.

Cremophor EL head portion consists of polyethylene glycols and glycerol ethoxylates, whereas the tail portion is composed of oxyethylated triglycerides of a long chain (C18) unsaturated ricinoleic acid, which is structurally similar to the long chain (C18) of unsaturated α-linolenic acid.

Both Tween 80 and Cremophor EL have a hydrophobic tail of similar length (C18) to α-linolenic acid. It is possible that these hydrophobic chains were responsible for increasing the solubility profile for α-linolenic acid. Both Tween 80 (Hydrophilic-Lipophilic Balance; HLB = 15) and Cremophor EL (HLB = 14) have high hydrophilic nature and good emulsion forming capacity due to their high HLB values. However, α-linolenic acid solubility was higher in Cremphor EL as compared to Tween 80. This could be because Tween 80 consists of a single chain of oleic acid as lipophilic part while Cremophore EL has three fatty acid chains attached to PEG-glycerol. This bulkier lipophilic part enhances the emulsification properties of Cremophore EL [20]. Nonetheless, Tween 20 (HLB = 16.7) showed low solubility capacity despite having high HLB value. This could be due the different hydrophobic tail group, which is composed of saturated medium-chain lauric acid (C12). These results are in agreement with the results of the previous study conducted by Mosca et al. [21] and show that the length and size of the hydrophobic side chains of surfactants determines the interactions with the oil phase [21]. Labrasol (HLB = 12), labrafil M2125 (HLB = 4) and Caproyl 90 (HLB = 6) showed the lowest solubility enhancement for α-linolenic. This might be due to their low HLB values that cause incompatibility between these hydrophilic surfactants and α-linolenic acid.

Based on saturation solubility studies, Transcutol P was selected as co-surfactant because α-linolenic acid showed good solubility in this solvent. A blend of surfactants and co-surfactant is needed to increase drug solubility and to obtain a stable microemulsion [22]. These co-surfactants help to further reduce the surface tension and fluidize the surfactant film, which increases the entropy of the system leading to its thermodynamic stability. Co-surfactants also increase the flexibility of the interfacial film around the microemulsion droplet [23,24].

It is known that a single surfactant is not sufficient to form balanced microemulsions and a combination of surfactant and co-surfactant is required to optimize the formation of a microemulsion [25]. Therefore, the selection of surfactant/co-surfactant blends is important in the formulation of a stable dispersion system.

The selected surfactants were blended with the selected co-surfactant in ratios of 1:1, 1:4 and 4:1 (*w*/*w*). Among the surfactant/co-surfactant blends, two mixtures had the highest solubilisation capacities for α-linolenic acid: Tween 80/Transcutol P 1:1 (*w*/*w*) and Cremophor EL/Transcutol P 4:1 (*w*/*w*). Thus, these two blends were selected to study the phase behaviour of α-linolenic acid.

### 2.2. Construction of Pseudo-Ternary Phase Diagrams

**Figure 2:**
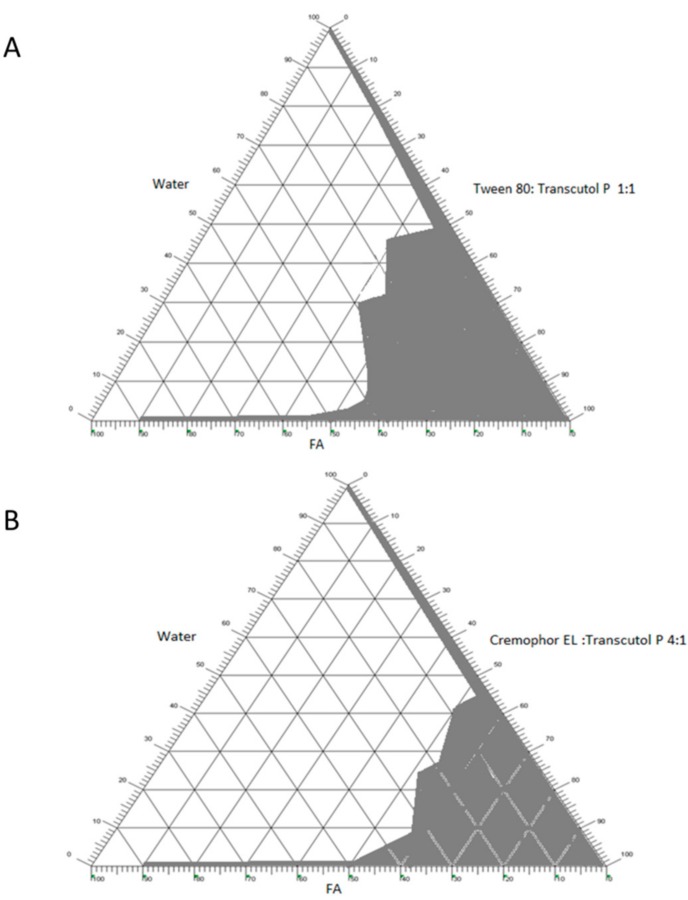
Pseudo-ternary phase diagrams of α-linolenic acid, water, surfactant/cosurfactant (S/CoS) mix made of (**A**) Tween 80/Transcutol P (1:1), (**B**) Cremophor EL/Transcutol P (4:1).

The pseudo-ternary phase diagrams were constructed using phase diagram by micro-plate dilution (PDMPD) method. Ternary phase diagrams were constructed by taking 1:1 ratio of Tween 80/Transcutol P and 4:1 ratio of Cremophor EL/Transcutol P. The shaded area of the phase diagrams shows the ME region, whereas the non-shaded area displays the none ME region. The pseudo-ternary phase diagrams of the α-linolenic acid/surfactant:co-surfactant/water are shown in Figure 2. The phase behaviour study revealed that the emulsion region was larger with 1:1 Tween 80/Transcutol P (Figure 2A) in comparison with Cremophor EL/Transcutol P (Figure 2B). From the phase diagrams, it can be observed that ME regions increase with increasing surfactant:co-surfactant mix ratio. This could be due to the effect of the hydrophilic co-solvents and/or co-surfactants that reduce the interfacial tension and increase the fluidity of the oil-water interface and hence, promoted the formation of microemulsions [23,24].

### 2.3. Preparation and Characterization of Microemulsions

Formulations were developed based on the microemulsion zone of pseudo-ternary phase diagrams. The composition of selected ME formulations is given in Table 1. All the developed formulations were found to be clear/transparent on visual inspection. The clarity of microemulsions was also checked by transparency, measured as percent transmittance (%T). All ME formulations showed % transmittance value greater than 98% (Table 1). These results indicate t that these compositions are isotropic one-phase systems.

#### 2.3.1. Characterization of Microemulsions

**Figure 3:**
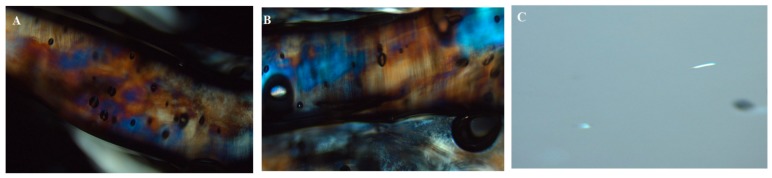
Photomicrographs of samples under polarized light microscopy (**A**) Lamellar liquid crystals of Tween 80/water, (**B**) Lamellar liquid crystals of Cremophor EL/water, (**C**) Microemulsion.

Polarized light microscopy can distinguish between isotropic and anisotropic materials. The ME samples containing water/surfactant:co-surfactant/oil were examined under polarized light for sample anisotropy and birefringence. The binary systems containing only water/surfactant were also observed under polarized light. The ME samples did not show any birefringence and appeared completely dark under polarized light (Figure 3C) characteristic of isotropic material. On the other hand, textures characteristic of lyotropic lamellar liquid crystals (were seen with water/surfactant micrographs as shown in Figure 3A,B. These observations indicate that all the ME formulations were optically isotropic colloidal dispersions.

The droplet size plays a significant role in the microemulsion stability and performance because it determines the rate and extent of drug release as well as in vivo absorption. It has been reported that the smaller droplet size of the emulsion provides better drug absorption by increasing the interfacial area in contact with biological membranes [26,27]. Table 1 shows the results of particle size analysis, polydispersity index (PDI) and zeta potential of prepared microemulsions. All ME formulations showed an average droplet size of more than 200 nm except T1 which showed the smallest droplet size of 190.4 ± 2.31 nm. The overall higher droplet size could be due to the fact that both surfactants used in ME formulations, Tween 80 and Cremophor EL, have alkyl carbon chain lengths of 18, which is similar to the long chain (C18) of unsaturated α-linolenic acid (oil). This finding was consistent with a previous study which reported that oil with carbon chain length similar to that of surfactant increases the average droplet size [28,29,30]. The PDI value for all formulations was less than 1 which is desirable (Table 1). The lower PDI value indicates a higher uniformity of the droplet size in the formulation [31]. Zeta potential is related to the stability of colloidal dispersions. Zeta potential indicates the degree of repulsion between adjacent, similarly charged particles in dispersion. When the zeta potential value is high, the electrostatic repulsive forces between the droplets increase which prevents the coalescence of the droplets. So, colloids with high zeta potential (negative or positive) are electrically stabilized [32,33]. The mean zeta potential value of the prepared microemulsions was consistently negative and ranged between −0.025 ± 0.011 and −0.394 ± 0.035 mV, which was generally of smaller magnitude (Table 1). These low zeta potential values could be due to the larger droplet sizes. The use of non-ionic surfactants, Tween 80 and Cremophor EL also lowers the zeta potential values [34]. Negative zeta potential measurements indicate that the interface is negatively charged. This negative charge imparts stability to the ME system by producing electrostatic repulsive forces of head groups which thereby hindering aggregation with nearby droplets [35].

The pH values of all ME formulations are given in Table 1. The pH of all ME formulation ranged between 4.66 and 6.23, which is within the normal pH range of marketed ophthalmic solutions. The normal pH value is one of the formulation considerations that may help reducing the eye irritation produced upon instillation.

All microemulsion samples were found to have viscosities in the range of 56 to 101 mPa∙S. Table 1 gives the viscosity measurements for all ME formulations. These results indicate that formulation C2 showed the highest viscosity. It was observed that there was an increase in viscosity with an increase in the water content and surfactant:co-surfactant ratio.

The drug content in the ME formulations was measured using the previously published Gas-chromatography (GC) method [17]. The percent drug content of all ME formulations is shown in Table 1. The amount of the FA in all ME formulations ranged from 92% to 99% of the original amount which indicates that the FA is stable when loaded into these ME systems.

**Figure 4:**
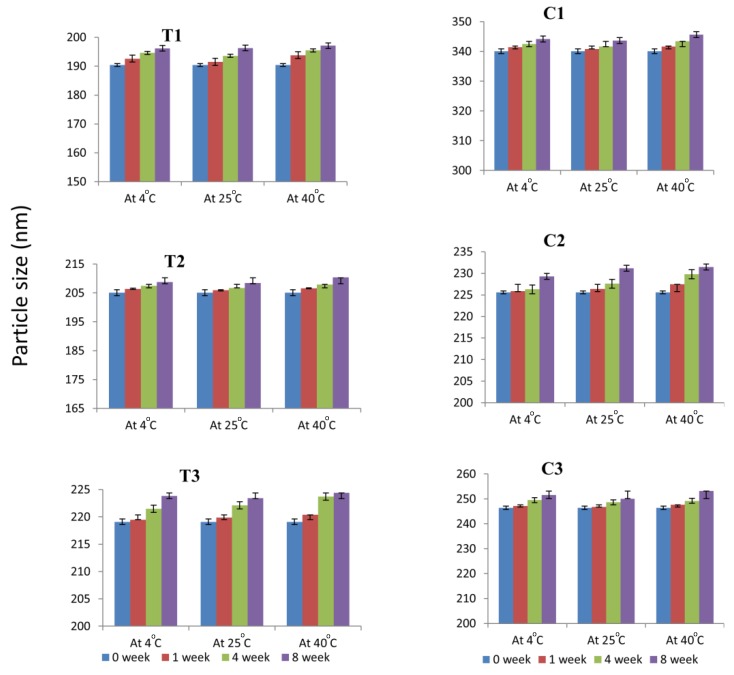
Particle size of microemulsion formulations at 0, 1 week, 4 weeks and 8 weeks interval at 4 °C, 25 °C and 40 °C. Mean ± SD, *n* = 3.

**Figure 5:**
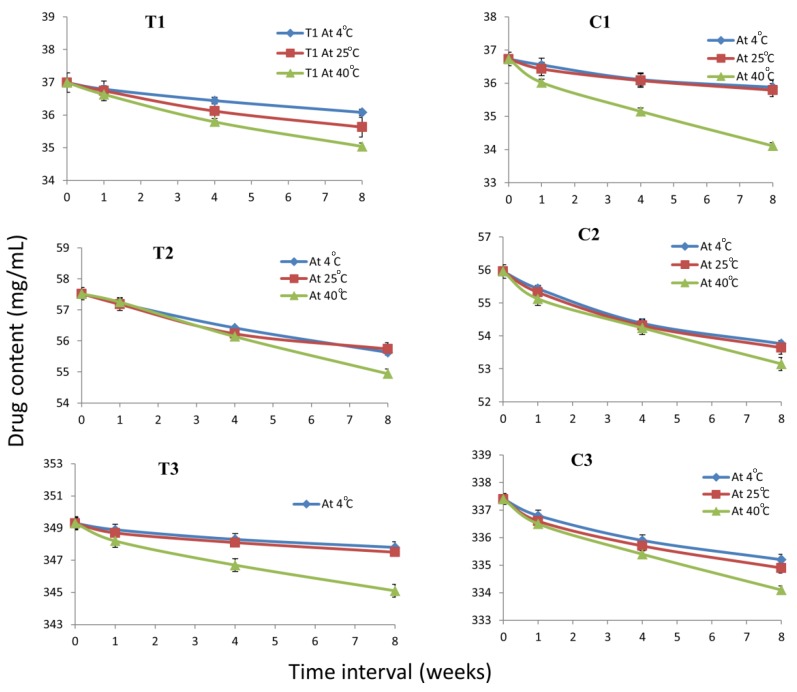
Drug content of microemulsion formulations after 8 week storage at various temperatures. Mean ± SD, *n* = 3.

To evaluate the stability of the selected ME formulations, they were kept at different temperatures (4 °C, 25 °C and 40 °C) for 8 weeks and were evaluated periodically. Results of stability studies indicated that after 8 weeks, there were no significant changes (*p* value > 0.05) in the initial droplet size and drug content of the microemulsions stored at 4 °C and 25 °C and there were no sign of phase separation or drug precipitation on storage. However, slight changes were observed in the initial droplet size and drug content of the microemulsions stored at 40 °C. This might be due to the oxidative degradation of the linolenic acid in the MEs at higher temperatures as suggested earlier by [36]. The results are shown in Figure 4 and Figure 5. Earlier reports [37] suggested the autoxidation of linolenic acid into hydroperoxides at carbon 9, 12, 13 and 16. Also secondary oxidation products such as propanal, 2-butenal and 2-pentenal were reported. The relative autoxidation rate of linolenic acid ranges between 1:40 and 50:100 according to the oxygen update [37]. According the current results it is evident that the stability of linolenic acid in the microemulsion formulations is high. Possibly the ME formulations reduced the oxygen contact with the fatty acid, hence protected the FA from degradation caused by autoxidation.

#### 2.3.2. Contact Angle Measurements

Therapeutic activity of the ophthalmic microemulsions depends on the extent to which the fluid wets and covers the corneal surface. Spreading is a fundamental phenomenon in which the fluid phase is displaced completely or partially on the surface of a solid. One of the most useful parameters that is typically used to describe spreading and wetting properties of ophthalmic MEs is the contact angle of the liquid on the hydrophilic corneal surface [38]. The surface of the cornea is typically covered with a hydrophilic mucoid or mucin layer of the tear film to transform the hydrophobic corneal surface into a hydrophilic surface [39]. Therefore, an ophthalmic microemulsion needs to both wet and spread on the corneal surface and then penetrate to maximize the therapeutic activity. The contact angle depends on surface tension of the liquid, surface free energy (interfacial tension between the liquid and the solid) and the interaction forces between the liquid and solid surface and between the liquid molecules themselves. If adhesive forces between the solid surface and the liquid are stronger, the droplet will completely spread out on the solid surface resulting in smaller contact angle. If the cohesive forces within the liquid drop (i.e. hydrogen bonds and Van der Waals forces) are stronger, the droplet will avoid contact with the solid surface resulting in larger contact angle [40]. If the contact angle is low, the fluid will spread to cover or “wet” a larger area of the surface. If the contact angle is high, the fluid will form a compact, self-contained droplet on the surface [41]. The results of contact angle measurements are given in Table 1. The contact angle of all ME formulations ranged from 12.2° to 25.2° on hydrophilic surface and from 25.9° to 43.8° on hydrophobic surface. These results indicate that all MEs had relatively low contact angle demonstrating good spreading ability. These results also revealed that all MEs possessed larger contact angle on hydrophobic surface as compared to hydrophilic surface. This could be due to the fact that interfacial tension is high on a hydrophilic surface and low on a hydrophobic surface. The high interfacial tension causes low interaction between the hydrophobic solid surface and ME droplet (low surface energy) which results in high contact angle, hence the poor spreading ability.

Overall results indicate that contact angle values for all MEs did not exceeded 90° on both hydrophilic and hydrophobic surfaces (Table 1), confirming that all MEs have the ability to cover and wet the ocular surface which could potentially translate to increased therapeutic effect.

#### 2.3.3. Self-Diffusion NMR

Self-diffusion NMR spectroscopy (DOSY) is a powerful technique for characterisation of microemulsion structures. It is used to distinguish between bicontinuous and droplet type microemulsions [42,43]. It utilizes apparent translational diffusion coefficients in characterizing the microemulsions [44,45]. The diffusion coefficients (*D*) were obtained from the slope of the equation:

(1) $$\ln\left(\frac{I_{g}}{I_{0}}\right)=-{[\gamma }^{2}g^{2}\delta^{2}\left(\Delta -\frac{\delta }{3}\right)]D$$

The characteristic NMR peaks for fatty acid (α-linolenic acid), surfactants (Tween 80 and Cremophor EL), Transcutol P and water. The proton signal of the terminal methyl group of fatty acid, surfactants and Transcutol P appeared at around 1.02 ppm, 0.95 ppm and 1.25 ppm, respectively, whereas a small peak at approximately 4.7 ppm assigned to water.

**Figure 6:**
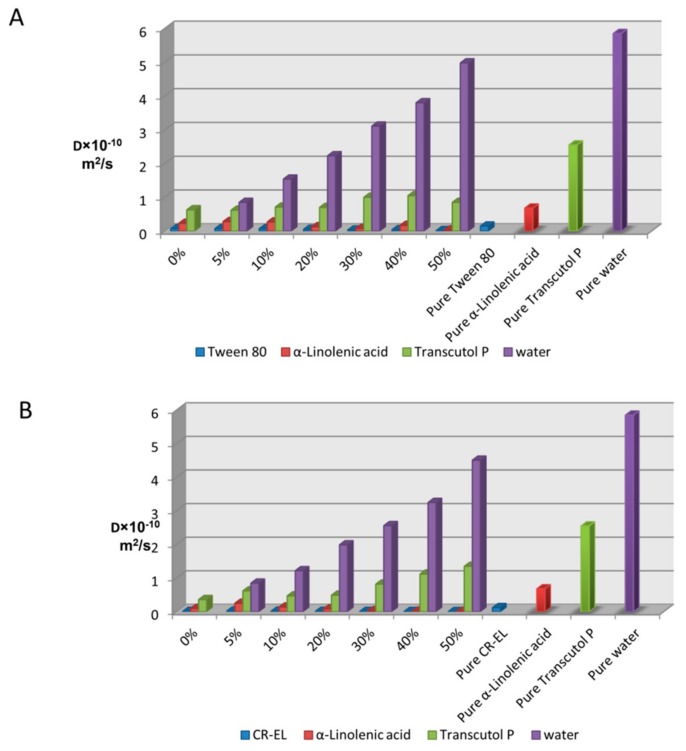
The self-diffusion coefficients of each component in α-Linolenic acid/water/Tween 80:Transcutol P(1:1) systems containing various concentrations of water from 0 to 50% (*w*/*w*) (**A**) and each component in α-Linolenic acid/water/CR-EL:Transcutol P(4:1) systems containing various concentrations of water from 0 to 50% (*w*/*w*) (**B**).

The self-diffusion coefficients of fatty acid, surfactants (Tween 80 and Cremophor EL), Transcutol P and water in ME systems were around 10^−1^ m^2^/s as shown in Figure 6A,B. These low self-diffusion coefficient values indicate that the bicontinuous microemulsions were not likely to have formed. The self-diffusion coefficients of pure components were also calculated as shown in Figure 6A,B. Then the self-diffusion coefficient values of components in all ME samples were compared with that of the pure component to determine the microemulsion type [42,43].

If the ME system is of a droplet-type, the self-diffusion of the components of the internal pseudo-phase is determined by the diffusion of the droplet itself and therefore will be slower than that of the pure components. In a bicontinuous microemulsion, where both oil and water are forming larger domains, the diffusion of both components is high and of the same magnitude as that of the pure components [42,43].

These results indicate that self-diffusion coefficients of fatty acid, surfactants, Transcutol P and water in the MEs were lower than that of the pure components. These results suggest the formation of droplet type microemulsions as there is no evidence on the presence of a bicontinuous microstructure from the current NMR data. These results also indicate that the self-diffusion coefficient of fatty acid and surfactants further decreased upon increase of water concentration. The self-diffusion coefficient of water was higher than that of α-linolenic acid, Tween 80, Cremophor EL and Transcutol P in all systems but a stronger increase in the water self-diffusion coefficient started at samples containing 30% *w*/*w* of water or more. This suggests a possible change from water-in-oil to oil in water at this point. 

#### 2.3.4. Bovine Corneal Opacity and Permeability (BCOP) Test 

**Figure 7:**
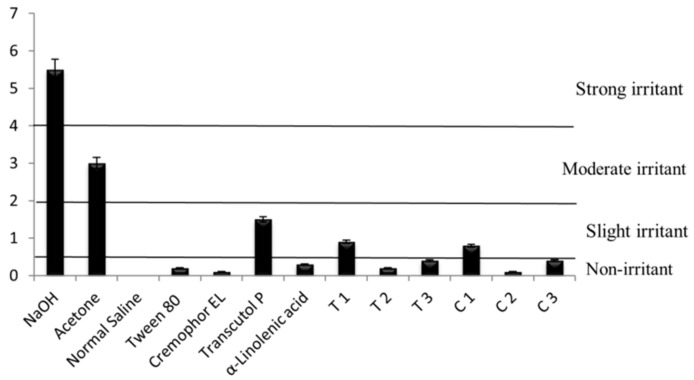
Cumulative BCOP scores of NaOH (strong positive control), Acetone (moderate positive control), Normal Saline (negative control), Tween 80, Cremophor EL, Transcutol P, and the microemulsion formulations; T1, T2, T3 and C1, C2 and C3. Results are expressed as mean values ± SD, *n* = 3.

The bovine corneal opacity and permeability (BCOP) test measures changes in corneal opacity, determined by changes in light transmission and permeability, measured by increases in permeability to fluorescein, as a result of exposure to a test substance. The opacity and permeability values are used to calculate an in vitro score, in order to reflect the ocular irritation potential [46]. This test is well suited to identify substances moderately and severely irritating to the eye [47]. Figure 7 shows the cumulative bovine eye test scores for the controls and test substances. Apart from Transcutol P and formulation T1 and C1, the average cumulative scores calculated for individual components (Tween 80, Cremophor EL and α-linolenic acid) and MEs (T2, T3, C2, C3) were found to be less than 0.5 (<0.5) indicating that they are non-irritating. These test substances did not show any signs of corneal injuries with no changes in corneal opacity, corneal permeability, or epithelial damage]. Transcutol P and ME formulation T1 and C1 with an average cumulative score of 1.5, 0.8 and 0.8, respectively, indicated minor irritation based on corneal opacity and fluorescein permeability.

**Figure 8:**
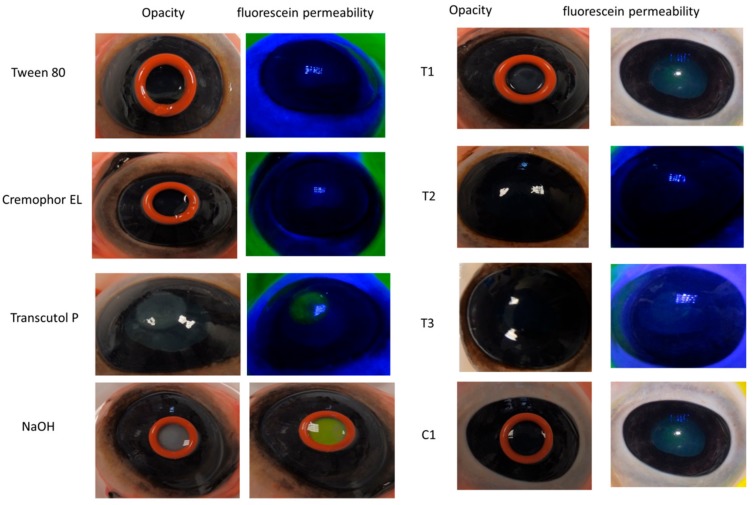
Degree of corneal opacity and fluorescein permeability for Tween 80, Cremophor EL, Transcutol P, NaOH and the microemulsion formulations; T1, T2, T3 and C1.

The healthy cornea is transparent and completely impermeable to fluorescein dye, due to the exclusive tight junctions of the corneal epithelium. Figure 8 show photographs of corneal opacity and fluorescein permeability for controls and test materials. These results showed that NaOH induced marked opacity and complete fluorescein staining of cornea whereas saline (negative control) caused no corneal opacity or staining as shown in Figure 8. Transcutol P caused slight irritation manifested as weak corneal opacity and permeability. Two formulations (T1 and C1) also exhibited slight corneal opacity and fluorescein staining which could be due to the high percentage of surfactant:co-surfactant in T1 and C1 (88% and 86%, respectively). Overall, the tested formulaions did not cause any strong corneal irritation signs and as such were regarded as safe to further test in live animals.

#### 2.3.5. Hen’s Egg Test Chorioallantoic Membrane (HET-CAM)

**Figure 9:**
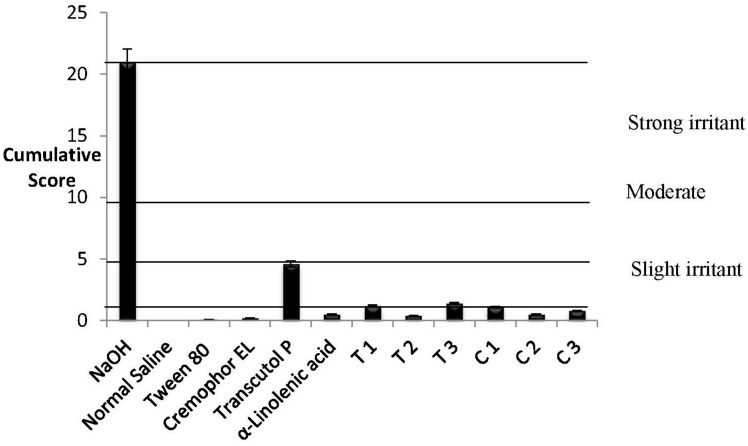
Cumulative HET-CAM scores of NaOH, Acetone, Normal Saline, Tween 80, Cremophor EL, Transcutol P, and the microemulsion formulations; T1, T2, T3 and C1, C2 and C3. Results are expressed as mean values ± SD, *n* = 3.

The HET-CAM provides invaluable information on the conjunctival irritation potential of the test substance. The CAM responds to injury with a complete inflammatory process similar to that induced by the conjunctival tissue of the eye [46]. After the treatment, the surface of the CAM was observed for any changes at different time points (after 30 s, 1 min, 2 min and 5 min) and the average cumulative HET-CAM test scores for the controls and test materials were calculated (Figure 9). The average cumulative scores calculated for individual components (Tween 80, Cremophor EL and α-linolenic acid) and MEs (T2, C2, C3) were found to be less than 0.9 (<0.9). These results reveal that these tested substances are practically non-irritant when applied to the surface of the CAM. In contrast, Transcutol P and three ME formulation (T1, T3 and C1) showed slight irritant effects with an average cumulative score of 4.6, 1.2, 1.4 and 1.1, respectively (Figure 9). The results of the ocular irritation of Transcutol P are in agreement with the findings of Liu et al. who found that Transcutol P causes slight irritation at a concentration of 0.05% without any visible ocular damage or abnormal clinical signs involving the cornea, iris, or conjunctivae at all concentrations [48].

**Figure 10:**
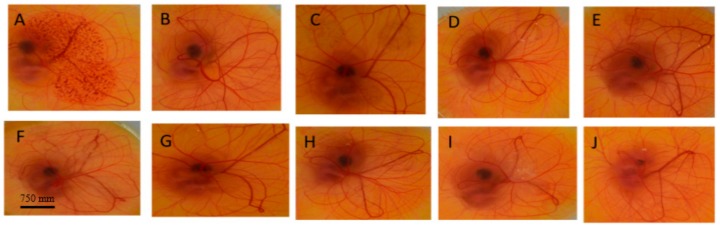
Effect of controls and test substances on the surface of the chorioallantoic membrane (CAM) after treatment for 5 min (**A**) NaOH, (**B**) Normal saline, (**C**) Transcutol P, (**D**) Tween 80, (**E**) microemulsion (T1), (**F**) microemulsion (T2), (**G**) microemulsion (T3), (**H**) microemulsion (C1), (**I**) microemulsion (C3), (**J**) microemulsion (C2).

Figure 10 shows the effects induced by the tested formulations and the selected controls on the surface of the CAM before and after treatment for 5 min of contact. These results showed that NaOH (positive control) induced major damage and caused complete lysis and degradation of immature blood vessels when applied to the surface of the CAM (Figure 10A), whereas the normal saline showed no effect (Figure 10B). Transcutol P caused slight degradation of blood vessels (Figure 10C). Three microemulsion formulations (T1, T3 and C1) developed minimal irritation potential manifested as very slight lysis of blood vessels after 5 min as shown in Figure 10E–G) respectively. Overall, none of the tested formulations caused any major damage to the blood vessels. These results indicate that all of the tested formulations did not show strong irritant effect when applied to the surface of the CAM.

#### 2.3.6. Antibacterial Activity of Microemulsions against *N. gonorrhoeae* and *S. aureus*

In this study, the individual components (α-linolenic acid, Tween 80, Cremophor EL and Trascutol P) and six formulated MEs were tested against *N. gonorrhoeae* and *S. aureus* (Table 2). The results showed that both *N. gonorrhoeae* and *S. aureus* were susceptible to all ME formulations. Fatty acids are known to kill or inhibit the growth of bacteria by disrupting cell membrane which is caused by interference with the electron transport chain and the disruption of oxidative phosphorylation. The electron transport chain is located in the inner membrane of bacterium and is essential source of energy for bacterium. The disruption of electron transport chain is caused either by directly binding of FAs to the electron carriers of the electron transport chain or by insertion into the inner membrane so the ability of the electron transport chain to transfer electrons is impaired. This result in reduced proton gradient and membrane potential which results in reduced ATP production, an essential source of energy for bacterium. FAs may also inhibit the bacterial growth by cell lysis, inhibition of enzyme activity, impairment of nutrient uptake and the generation of toxic peroxidation and autoxidation products [49]. Among the individual components Tween 80, Cremophor EL and Transcutol P exhibited antibacterial activity against only *S. aureus*. These results also indicate that T3 (35% α-linolenic acid and 60% surfactant:co-surfactant) and C3 (35% α-linolenic acid and 60% surfactant:co-surfactant) exhibited the larges antibacterial zones of inhibition against both microbial agents compared to other formulations. T1, T2, C1 and C2 showed medium antibacterial zone against *S. aureus* whereas these MEs showed lowest antibacterial zone against *N. gonorrhoeae*. Overall, all ME formulations showed strong inhibitory effect against *S. aureus* compared to *N. gonorrhoeae*. This might be due to the fact that all excipients (Tween 80, Cremophor EL, Transcutol P) showed strong antimicrobial effect against *S. aureus* compared to *N. gonorrhoeae*.

## 3. Materials and Methods

### 3.1. Materials

Tween 80, Tween 20, Transcutol P, Cremophor EL, PEG 400 and α-linolenic acid were purchased from Sigma (Sigma Aldrich, Dorset, UK). The derivatisation reagent, BCl_3_-methanol 12% *w*/*w* (12% boron trichloride in methanol), n-hexane (HPLC grade, purity, ≥99%) were also purchased from Sigma (Sigma Aldrich, Dorset, UK). Labrasol, Capryol 90 and Labrafil M2125 were kindly gifted from Gattefosse Company (Bracknell, UK).

### 3.2. Microorganisms

Fresh cultures of *N. gonorrhoeae* strain NCCP11945 were grown on GC agar (Oxoid, Basingstoke, UK) at 37 °C in an atmosphere of 5% CO_2_ for 24 h. The colonies were removed from the culture plate with a loop and suspended into a 3 mL GC broth until cloudy. The culture was mixed well and standard density was adjusted to 10^7^ CFU per mL. 

Fresh cultures of *S. aureus* strain NCTC06571 were grown on nutrient agar (Oxoid, Basingstoke, UK) at 37 °C for 24 h. The colonies were removed from the culture plate with a loop and suspended into a 3 mL Ringer solution until cloudy. The culture was mixed well and the standard density was adjusted to 0.5 McFarland (1.5 × 10^8^ CFU per mL).

### 3.3. Preformulation Studies

#### 3.3.1. Excipient Selection (Surfactants & Co-Surfactants)

The selection of suitable excipients is essential in the development of microemulsion. Excipients having maximum solubilizing potential for the fatty acid are selected for the formulation of the α-linolenic acid based ME. Selection of excipients was done by reviewing the published literature. Surveying the literature, a range of excipients was checked for use in ophthalmic preparations. Tween 20, Tween 80, Cremophor EL, Capryol 90, Labrasol and Labrafil M2125 were selected as surfactants and Transcutol P and PEG 400 were selected as co-surfactants.

#### 3.3.2. Determination of Saturation Solubility of α-Linolenic Acid in Different Surfactants and Co-Surfactants

The solubility of α-linolenic acid in various surfactants and co-surfactants was determined using a 96 well plate method reported by Bharate et al. [50]. Stock solutions of fatty acid were prepared in methanol (1 µg/mL to 1800 µg/mL). 250 µL of each of stock solution was transferred into the 96-well plates and the solvent was evaporated then 250 µL of different surfactants and co-surfactants was added into the wells and plates were shaken horizontally at 600 rpm for 5 h at room temperature. The plates were then centrifuged at 3000 rpm for 15 min and then samples were analysed by UV spectrophotometer at 350 nm. Each experiment was performed in triplicate. The solubility of α-linolenic acid was also determined in different ratios of surfactant and co-surfactant as 1:1, 1:4 and 4:1 using 96 well plate method reported above.

#### 3.3.3. Selection of Surfactant & Co-Surfactant Blend

Based on the individual solubility studies, Tween 80 and Cremophor EL were selected as surfactants and Transcutol P was selected as co-surfactant. The solubility of α-linolenic acid was also determined in various surfactants and co-surfactants mixtures. The individual non-ionic hydrophilic surfactant was blended with the selected co-surfactant in ratios of 1:1, 1:4 and 4:1 (*w*/*w*).

#### 3.3.4. Construction of Pseudo-Ternary Phase Diagram

The pseudo-ternary phase diagrams were constructed to determine the concentration range of all components (α-linolenic acid/surfactant/co-surfactant/water) in which they form a microemulsion. The surfactant and co-surfactant were mixed at 1:1 and 4:1 ratio. Different mixtures of α-linolenic acid and surfactant/co-surfactant mixtures were prepared at weight ratios of 0:10, 0.5:9.5, 1:9, 2:8, 3:7, 4:6, 5:5, 6:4, 7:3, 8:2 and 9:1, respectively. The pseudo-ternary phase diagrams were constructed by using the phase diagram by micro-plate dilution (PDMPD) method, a novel technique based on the water titration method [51]. The microtitre plates were filled by pipette in accordance with the filling scheme: The wells A1 to D5 were filled in two steps. Fatty acids/surfactants/co-surfactants mixture was filled into the wells with 200 μL at the first well and down by increments of 5 μL to reach 0 μL. Then, water was added to each well to make a final volume of 200 μL. After the plates had been filled, they were sealed and then shaken for 24 h at room temperature (25 °C). After that, the microplates were characterised by measuring absorbance using microplate reader at 600 nm and by making a visual evaluation of the isotropy and the border between the homogeneous or the heterogeneous system.

The percentage of transmittance of the microemulsion formulations was determined using the following formula, where T is transmittance and A is absorbance.


%T = antilog (A − 2)
(2)

#### 3.3.5. Preparation of Microemulsion Formulations

According to microemulsion region in the phase diagram, ME formulations were selected at different component ratios. α-linolenic acid (ALA) was used as an oil phase. Tween 80 and Cremophor EL were selected as surfactants. Transcutol P was selected as co-surfactant with water as aqueous phase. Surfactant and co-surfactant were mixed at different mass ratios (1:1, 4:1). α-linolenic acid was dissolved under stirring in mixture of S/CoS. Then, the appropriate amount of water was added to the mixture drop by drop with continuous stirring.

#### 3.3.6. Characterization of Microemulsions

##### Visual Evaluation

Microemulsions were first monitored for transparency, signs of phase separation and birefringence with the aid of visual evaluation. Visual evaluation helps to differentiate between microemulsions and other two phase systems such as emulsions. Visually microemulsions are transparent or translucent, whereas emulsions are turbid.

After visual inspection, the formulations which have better clarity and no phase separation were confirmed for selection as clarity of the formulation is the initial priority of the microemulsion [52].

##### Polarized Light Microscopy

Plane polarized light microscopy was used to distinguish between pure microemulsions and microemulsions containing lamellar liquid crystals. ME samples were prepared by placing a drop of ME between a coverslip and a glass slide and were then examined using cross-polarized light microscopy (Polarizing Microscope RPL-55 Series, Radical Instruments, Ambala Cantt, India). The isotropic and anisotropic behaviour of the samples was observed. Isotropic materials such as microemulsion, in contrast to anisotropic liquid crystals, do not interfere with the polarized light and the field of view remains dark because the analyser absorbs light passing through the polarizer. 

##### Droplet Size and Zeta Potential Measurement

The droplet size, polydispersity index (PDI) and zeta potential (ZP) of MEs were measured by dynamic light scattering using a Zetasizer (Malvern instruments Ltd., Malvern, UK). ME samples were analysed in triplicate at 25 °C.

##### Determination of pH

The pH values for microemulsions were determined at 25 °C by pH meter (JENWAY model 3305, JENWAY Ltd., Stone, UK). All measurements were carried out in triplicate.

##### Viscosity Measurements

Viscosity of samples was measured at 25 °C with a Brookfield viscometer (DV-II+Pro Brookfield., Middleboro, MA 02346, USA) using spindle No. 34. With shear rate 50 rpm. Each measurement was performed in triplicate.

##### Drug Content Determination

Concentration and drug content of FA-based ME formulations were determined using the Gas Chromatography-Flame Ionization Detector (GC-FID) method [17]. ME samples were derivatised by using BCl_3_-methanol (12% *w*/*w*) in a water bath at 60 °C for 8 min. Then, samples were extracted with n-hexane (1–2 mL) by hand-shaking for 1 min until both layers were clear. The layers were allowed to settle and the upper (organic) layer was transferred into a clean vial. The organic layer containing fatty acid methyl esters (FAMEs) was dried by adding 500 mg of anhydrous sodium sulphate. Then, samples were analysed by the GC-FID method [17].

##### Contact Angle Measurements

Contact angles of prepared microemulsions were measured with goniometer method [40]. A 5 μL droplet of the microemulsion was placed on the surface of a plate and the image was immediately sent via the CCD camera to the computer for analysis. Contact angles were determined as the cosine (θ) of the contact angle (θ) between the ME droplet attached to the hydrophilic surface (dry glass slide) or hydrophobic surface (glass slide covered with parafilm) and the droplet. For all tests, the mean value of at least three replicate evaluations was reported.

#### 3.3.7. Stability Studies

Physical and chemical stability testing of selected microemulsions were performed under the accelerated conditions in triplicate (*n*  =  3) to find out the stable microemulsions. For stability testing, microemulsions were kept at various temperatures (4 °C, 25 °C and 40 °C) for 8 weeks. The clarity, phase separation, particle size, zeta potential and drug content of tested microemulsions were determined at 0, 1 week, 4 weeks and 8 weeks [53].

#### 3.3.8. Self-Diffusion NMR

Self-diffusion NMR measurements were carried out at 600 MHz using a Bruker Avance DRX 600 (Billerica, MA, USA) at 25 °C. Each sample was dissolved in D_2_O (internal standard) and filled into NMR tube. The self-diffusion coefficients of pure components were calculated. The self-diffusion coefficients of components in the microemulsion samples were compared with those of the single components to determine the type of microemulsion. The diffusion coefficients (*D*) were obtained from the slope of the Stejskal-Tanner equation [54]:

(3) $$\ln\left(\frac{I_{g}}{I_{0}}\right)=-{[\gamma }^{2}g^{2}\delta^{2}\left(\Delta -\frac{\delta }{3}\right)]D$$

where **I_g_** and **I_0_** are intensities of the NMR signal in the presence and absence of field gradient pulses; γ is the gyromagnetic constant for 1 H; *g* is the duration of the z-gradient pulse; δ is the gradient strength; and Δ is the time interval between the gradient pulses.

#### 3.3.9. Antibacterial Activity of MEs against *N. gonorrhoea* and *S. aureus*

The antimicrobial activity of prepared microemulsions and its individual components against *N. gonorrhoea* and *S. aureus* were checked using disc diffusion method [17,18]. Blank paper discs (6 mm diameter) were loaded with 10 μL of the microemulsion formulations and its individual components and allowed to air-dry at room temperature. Nutrient agar plates were inoculated with bacterial suspension by dipping a sterile cotton wool swab into the suspension and spreading the inoculum evenly over the entire surface of the plates by swabbing in three directions. Plates were allowed to dry before applying discs. Then, the discs containing the test agents were applied to the surfaces of inoculated plates. Plates were inverted and incubated at 37 °C for 24 h to allow for bacterial growth. Inhibition zone diameters were measured in millimetres. All measurements were carried out in triplicate.

#### 3.3.10. Ocular Irritation Testing

In this study, BCOP & HET-CAM tests were used to investigate the ocular irritation potential of prepared microemulsions and their ingredients.

##### BCOP Test

Bovine’s eyes acquisition and examination. Bovine eyes were obtained from a local slaughter house. The eyes were examined for epithelium detachment, corneal opacity and corneal vascularization. Eyes with corneal damage or abnormalities were discarded.

Test substances. NaOH (0.5 M) was used as a positive control strong irritant, acetone as a moderate irritant and normal saline as a negative control [46]. Microemulsions and all used ingredients (α-linolenic acid, Tween 80, Cremophor EL and Transcutol P) were investigated for their corneal irritation potential using BCOP test.

Irritation testing, scoring and classification. The eyes were held with small plastic cups (cornea upwards) in the humid atmosphere of a closed water bath at 37 °C + 0.5 °C for 10 min. A silicon O-ring was carefully placed on the central part of the cornea. One drop of saline was applied inside the ring and eyes were equilibrated in a closed water bath for 5 min. Then the test substance was applied to the cornea inside the ring at a volume of 0.1 mL. After 30 s, the eyes were rinsed with approximately 10 mL saline and further incubated in the closed water bath for 10 min. The extent of corneal injury was assessed by evaluating the opacity. Then sodium fluorescein solution (2% *w*/*v* and pH 7.4) was applied to the cornea and corneal permeability was assessed using examination lamp and cobalt blue filter and following the scoring systems [46] in Table 3.

##### HET-CAM Test

Preparation of the CAMs. CAMs were prepared following the protocol early described by Reference [18]. Briefly, freshly collected fertilised hen’s eggs were incubated at 37.5 °C ± 0.5 °C and 66% ± 5% relative humidity (RH) for 3 days. During incubation, eggs were turned by hand five times per day to prevent the attachment of the embryo to one side of the egg. On day three, the eggshells were opened by cracking the underside of the egg against the edge of a plastic Petri dish. The content of the shell was then poured into a growing chamber. The growing chamber was made of a glass beaker over which a piece of cellophane membrane was attached and fixed in position using a circular plastic sleeve. Once in the growing chamber, each egg was examined for the viability of the embryo (intact CAM and yolk sac). Defective or non-viable eggs were discarded and only viable embryos with intact CAMs and yolk sacs were further incubated [46,55].

NaOH (0.1 M) was used as a positive control strong irritant and normal saline as a negative control. All prepared microemulsions and excipients; (Tween 80, Transcutol P, PEG 400, α-linolenic acid) were tested for their ocular irritation potential.

Irritation testing, scoring and classification. On day 10, 0.2 mL of the test substances was placed onto the CAM. For each test substance three eggs were used. After treatment with test substances, the blood vessels and capillaries were examined for the irritant effects of hyperaemia, haemorrhage, clotting and/or coagulation at different times post application [25,26]. A time dependent numerical score was then allocated to each test compound or formulation. The sum of the time dependent numerical scores for all three responses of hyperaemia, haemorrhage, clotting and/or coagulation gives a single numerical value (Table 4). The mean value of four tests allows for the assessment by a classification scheme similar to the Draize test [56].

#### 3.3.11. Statistical Analysis

All the experiments were repeated three times and data were expressed as the mean value ± SD. Statistical analysis was performed using the software Graphpad Prism (Graphpad Prism software, Inc., San Diego, CA, USA). Statistical data were analysed by one-way analysis of variance (ANOVA) and Student’s t test. Differences were considered significant for *p* < 0.05.

## 4. Conclusions

In this study, fatty acid based microemulsions were prepared and evaluated. The selection of surfactant and cosurfactants were selected after evaluating the saturation solubility of lineolenic acid in different systems while Pseudo-ternary phase diagrams were constructed to evaluate the regions of microemulsion. Nine different fatty acid-based microemulsions were prepared comprising of α-linolenic acid as oil phase, Tween 80 and Cremophor EL as surfactant, Transcutol P as co-surfactant and water as aqueous phase. The prepared microemulsions were characterized, showing an average particle size around 250 nm and a pH of 5.5. The prepared ME formulations were isotropic colloidal dispersions and did not show any lamellar and hexagonal liquid crystals when examined using polarized light microscopy.

All ME formulations were stable upon storage for 8 weeks without significant change in particle size and drug content as it is believed that the ME formulations protected the α-linolenic acid from autoxidation. All MEs exerted strong antimicrobial effects against *N. gonorrhoeae* and *S. aureus* without being irritant to the eye as suggested by the BCOP and HET-CAM studies. The study suggests that α-linolenic acid ME can be used as an effective and stable therapy for treatment of ophthalmia neonatorum conditions caused by *Neisseria gonorrhoeae* and *Staphylococcus aureus*.

## Figures and Tables

**Table 1:** Composition and characterisation criteria of the selected microemulsion formulations, fatty acid microemulsions were characterised for their particle size (nm), zeta potential (mV), light transmittance (%), viscosity (mPa∙S) and contact angle (θ). Data is presented as mean ± standard deviation (SD), where *n* = 3.

Formulation	Composition			Particle Size ± SD (nm)	PDI ± SD	Zeta Potential (mV)	% Transmittance (at 600 nm)	pH	Viscosity (mPa∙S)	α-Linolenic Acid Content %	Contact Angle on Hydrophilic Surface (^°^)	Contact Angle on Hydrophobic Surface (^°^)
	Fatty Acid (FA)%	S/CoS%	Water%									
T1 (Tween80/Transcutol P)	4	88	8	190.4 ± 2.3	0.309 ± 0.12	0.124 ± 0.022	98%	5.96 ± 0.02	65.32 ± 2.15	92.5	14.1 ± 0.85	29.5 ± 2.43
T2 (Tween8/Transcutol P)	6	60	34	205.1 ± 1.2	0.551 ± 0.085	0.107 ± 0.014	98%	4.66 ± 0.01	96.12 ± 4.22	95.9	12.2 ± 0.06	35.2 ± 3.73
T3 (Tween8/Transcutol P)	35	60	5	219.1 ± 1.5	0.383 ± 0.056	0.394 ± 0.035	115%	5.33 ± 0.02	56.67 ± 2.32	99.8	15.7 ± 2.51	25.9 ± 3.70
C1 (Cremophor EL/Transcutol P)	4	86	10	340.1 ± 1.9	0.561 ± 0.032	0.025 ± 0.011	99%	6.23 ± 0.02	82.53 ± 1.63	91.8	25.1 ± 2.41	34.8 ± 3.08
C2 (Cremophor EL/Transcutol P)	6	62	32	225.6 ± 2.1	0.392 ± 0.14	0.303 ± 0.054	98%	5.43 ± 0.01	101.42 ± 3.11	93.3	16.2 ± 3.36	43.8 ± 4.30
C3 (Cremophor EL/Transcutol P)	35	60	5	246.4 ± 3.3	0.484 ± 0.025	0.102 ± 0.012	97%	5.47 ± 0.02	74.46 ± 2.56	96.4	25.2 ± 2.60	38.0 ± 5.68

**Table 2:** Growth inhibition zone diameter of three selected α-linolenic acid based ME formulations and individual components against *N. gonorrhoeae* and *S. aureus* mean ± SD% (*n* = 3).

Formulation	Zone of Inhibition against *N. gonorrhoeae*	Zone of Inhibition against *S. aureus*
T1	6.5 ± 0.7 mm	14.5 ± 0.7 mm
T2	8.5 ± 0.7 mm	15 ± 1.00 mm
T3	22 ± 1.00 mm	21.5 ± 0.7 mm
C1	6.75 ± 0.4 mm	17.75 ± 0.4 mm
C2	8 ± 1.00 mm	16.2 ± 0.8 mm
C3	22.75 ± 0.4 mm	22.25 ± 1.06 mm
Active ingredients		
Tween 80	0.00	16.0 ± 1.4 mm
Cremophor–EL	0.00	11.5 ± 0.7 mm
Transcutol P	0.00	7.5 ± 0.7 mm
α-linolenic acid (1 mM)	10.2 ± 0.6	7.5 ± 0.6

**Table 3:** Bovine eye scoring system.

Opacity	Score	Fluorescein Permeability	Score	Cumulative Score	Interpretation
**None**	0	None	0	≤0.5	None
**Slight**	1	Diffuse and weak	0.5	0.6–1.9	Slight
**Marked**	2	Confluent and weak	1	2.0–4.0	Moderate
**Severe**	3	Confluent and intense	1.5	>4	Severe
**Opaque**	4	-	-	-	-

**Table 4:** Irritation scores and interpretations used in HET-CAM test.

	Score			Cumulative Score	Irritation Assessment
Effect	0.5 min	2 min	5 min	0–0.9	None
Hyperaemia	5	3	1	1.0–4.9	Slight
Haemorrhage	7	5	3	5.0–8.9	Moderate
Coagulation	9	7	5	9.0–21.0	Severe
